# Morphia; Hypodermically

**Published:** 1882-11

**Authors:** J. Graball


### MORPHIA; HYPODERMICALLY.

Dr. Sarchet asks in the *U. S. Investigator* of July 1st if he did right in refusing to give morphine and thereby losing a fee. Now to me it is a clear case that he did wrong. If he could make five dollars or five cents he ought to give morphine hypodermically, internally or externally. If you can get a patient, give him allopathic treatment if you can hold him thereby. What is the difference? You get the money which would have gone to an allopath, and by having two strings to your bow you can make money out of the allopathic patrons as well as the homœopathic ones. If you can't cure your patients with homœopathic treatment, give them quinine, but never fail to tell the people, in season and out of season, how much better your treatment is than that of the old school in chills. So give morphine for pain, quinine for intermittents, aconite for fevers, ergot for uterine hemorrhage, injections of carbolic acid for piles, cathartic pills for constipation, Fowler's solution for eczema, astringents for diarrhœa, etc., urethral injections for gonorrhœa, caustics for chancres, mercurial inunctions for constitutional syphilis, caustics and astringents for whites, forceps as often as possible in obstetrics. You will thereby show your skill in the use of instruments and probably give yourself a chance to operate for rupture of the perinæum—full fees, you know.

There is another advantage by these modes of treatment; it is much easier than it is to crowd your head full of symptoms and thus be a mere symptom coverer, which is so unscientific. True, all these expedients which some people call allopathic are followed by bad results to the patients. Well, we have collected the bills, impressed the people with our skill, and made a class of patients that, if we can only retain the impression we have made, will keep us busy through the rest of our lives.

And, moreover, did not the American Institute at its last meeting resolve that "It is the sense of the American Institute that no physician can properly sustain the responsibilities, or fulfill all the duties of his professional relations, unless he enjoys absolute freedom of medical opinion and unrestrained liberty of professional action, as provided in the code of ethics of this Institute?"

I hope I have convicted the Doctor of doing wrong.

J. Graball.

